# EEG-based multivariate and univariate analyses reveal the mechanisms underlying the recognition-based production effect: evidence from mixed-list design

**DOI:** 10.3389/fnhum.2025.1507782

**Published:** 2025-01-29

**Authors:** Bohua Zhang, Alhassan Abdullah, Minmin Yan, Yongqing Hou, Antao Chen, Helen McLaren

**Affiliations:** ^1^College of Education, Psychology and Social Work, Flinders University, Adelaide, SA, Australia; ^2^Faculty of Psychology, Southwest University, Chongqing, China; ^3^School of Social Work and Arts, Charles Sturt University, Thurgoona, NSW, Australia; ^4^Department of Neurobiology and Department of Psychiatry of the Second Affiliated Hospital of Zhejiang University School of Medicine, School of Brain Science and Brain Medicine of the Zhejiang University School of Medicine, Hangzhou, China; ^5^School of Psychology, Shanghai University of Sport, Shanghai, China; ^6^School of Allied Health (VIC), Australian Catholic University, Melbourne, VIC, Australia

**Keywords:** reading aloud, silent reading, LPC, FN400, MVPA

## Abstract

The production effect (PE) is a phenomenon where reading words aloud, rather than silently, during study leads to improved recognition memory. Human recognition memory can be divided into recollection (recognition based on complex contextual information) and familiarity (recognition based on a sense of familiarity). This study explored how reading aloud affects recollection and familiarity using electroencephalography (EEG) in a mixed-list design. Participants encoded each list item, either aloud or silently during the study phase and made remember/know/new judgments in the test phase, while EEG data were recorded. The behavioral results replicated the classic PE pattern and indicated that the PE was present in both recollection and familiarity. At the Event-Related Potential (ERP) level, the recollection-based LPC (late positive complex) old/new effect at test was largest in the aloud condition; however, the familiarity-based FN400 old/new effect was equivalent when comparing the aloud condition and the silent condition. Moreover, this study was the first to employ multivariate pattern analysis (MVPA) to decode the time course between two distinct memory strategies (aloud vs. silent). The results revealed significant decoding between 760 and 840 ms, which is consistent with the LPC old/new effect. The paper discusses both traditional theories and the Feature Space Theory based on our results, highlighting inconsistencies with assumptions regarding unconscious retrieval in the Feature Space Theory. In summary, the current results support the role of distinctiveness (enhanced memory for auditory or action information, consistent with recollection) in the PE, rather than the role of strength (enhanced memory trace, consistent with familiarity). This study suggests that enhanced distinctiveness/recollection may be a shared mechanism underlying certain advantageous memory strategies.

## 1 Introduction

Memory plays a crucial role in human learning and daily life, as it allows for retention and retrieval of information when needed. Consequently, people are always on the lookout for effective strategies to enhance their memory. Dunlosky et al. (2013) provides a comprehensive review of 10 common memory strategies, revealing that only a few may genuinely be effective (Dunlosky et al., 2013). However, one simple yet powerful strategy was notably absent from the discussion: reading aloud.

Memory associated with reading aloud is considered stronger than when silent reading (see MacLeod and Bodner, 2017; for a brief review). This effect was first reported by Hopkins and Edwards (1972), but it was only after MacLeod et al. (2010) delineated this phenomenon and coined the term, production effect (PE), that this effective encoding strategy received increasing attention from researchers. Since MacLeod et al. (2010), a substantial number of researchers have reported on PE (e.g., Bodner et al., 2020; Forrin et al., 2012; Kelly et al., 2024; Lin and MacLeod, 2012; López Assef et al., 2021; Saint-Aubin et al., 2021; Whitridge et al., 2024; Zormpa et al., 2019; Zhang et al., 2023a,b), especially in the recognition memory field (e.g., Bodner et al., 2014; Fawcett, 2013).

Research has primarily investigated the mechanism of PE (e.g., Bodner et al., 2016; Fawcett and Ozubko, 2016; MacLeod and Bodner, 2017; Ozubko and MacLeod, 2010). Up to now, the two dominant explanations of PE are the distinctiveness account and the strength account. In the distinctiveness account, MacLeod et al. (2010) emphasized that reading aloud involves unique phonological and articulatory processing, enabling participants to encode distinctive information during the encoding phase. During the recognition phase participants are said to retrieve this distinctive information, which facilitates recognition. Because the recognition process described in the distinctiveness account is based on the retrieval of contextual information, it aligns with recollection—the process of recognition memory associated with contextual information (Yonelinas et al., 2022). Therefore, by definition, recollection is consistent with distinctiveness (Fawcett and Ozubko, 2016; Zhang et al., 2023a). The strength account on the other hand emphasizes that reading aloud does not rely on retaining information about distinctiveness. Instead, it enhances the activation of memory traces for studied items, thereby increasing their familiarity during testing (Bodner and Taikh, 2012). This description aligns with the familiarity process in recognition memory (Yonelinas et al., 2022), where participants rely on the sense of familiarity for recognition decisions. Therefore, by definition, familiarity is inherently aligned with the concept of strength (Fawcett and Ozubko, 2016; MacKenzie and Donaldson, 2007; Parks, 2007; Yonelinas et al., 2022; Zhang et al., 2023a).

Previous studies have primarily employed two paradigms to explore the roles of distinctiveness and strength in the PE (Fawcett and Ozubko, 2016; Ozubko et al., 2012; Zhang et al., 2023a). The first is the mixed-list design, which is the most common and primary paradigm (MacLeod and Bodner, 2017). In this paradigm the vocabulary to be learned is often divided into two sets, possibly labeled as the blue set and the yellow set. Participants are required to read aloud or silently either the blue or the yellow font. The instructions represented by the two colors are counterbalanced across participant. Subsequently, during the studying phase, these items are randomly mixed (Bodner et al., 2016; MacLeod et al., 2010). The participants are then required to identify both studied and unstudied words. The second paradigm, which is less commonly used, is the block design. This design usually involves participants reading a series of words aloud continuously, followed by reading a series of words silently.

The size of the PE differs between the mixed-list design and the block design. Bodner et al. (2014) found that the PE in mixed-list designs is larger relative to that in the block design. This is because the memory of silent reading in the mixed-list design is worse than in the block design, showing a cost of silent reading. This cost may arise from lazy reading, wherein people tend to perceive the words read aloud as more important in the mixed-list. This leads them to reduce effort during the silent reading phase, consequently causing a decline in memory performance during silent reading (Bodner et al., 2014). The occurrence of this cost may be related to reading aloud interrupting covert rehearsal, which prevents participants from effectively maintaining the memory of words read silently after reading aloud (Cyr et al., 2022). An item-order account suggests that commonly processed items (silent reading) incur a cost in mixed-lists because the presence of unusually processed items (reading aloud) disrupts the encoding of relational information (Jonker et al., 2014).

At the mechanism level, greater PE at the behavioral level might be related to amplified distinctiveness or strength. A speculative piece of evidence comes from Ozubko et al. (2020), who explored whether adding a third condition could amplify the additional cost of silent reading. Their behavioral results indicated that in the group where the “important” condition (participants were instructed to remember the information carefully) was added, memory performance in the silent reading condition was worse when compared to the group without the “important” condition, showing a kind of cost. This cost is reflected in recollection (which is consistent with the definition of distinctiveness) and in familiarity (which is consistent with the definition of strength) (see Exp. 6; Ozubko et al., 2020). Moreover, Ozubko et al. (2020) proposed that this cost was due to participants looking for distinctiveness encoding information during the test. In conditions without distinctiveness encoding (silent reading), participants' confidence decreased. Regardless of the cause of the cost in the mixed-list, we can infer that the cost of silent reading might be reflected in recollection or familiarity, thereby potentially amplifying the role of distinctiveness or strength in the PE. This may ultimately lead to the larger mixed-list PE. Thus, if a block-based study only finds that recollection/distinctiveness contributes to the PE (see Zhang et al., 2023a), it is necessary to explore whether familiarity/strength also contributes to the PE in the context of a mixed-list.

Zhang et al. (2023a) used electroencephalography (EEG) technology to investigate the effects of reading aloud on recollection and familiarity. However, the results of their study need to be further examined using a mixed-list task (Zhang et al., 2023a). Initial research found that reading aloud can simultaneously enhance both recollection and familiarity at the behavioral level (see Fawcett and Ozubko, 2016: Dual processing account). However, ERP technology is a more sensitive method for exploring recollection and familiarity (Zhang et al., 2023a). In terms of ERP indicators of recollection and familiarity, the LPC component during the recognition phase varies with recollection, while the FN400 component varies with familiarity (Bridger and Mecklinger, 2012; Curran and Friedman, 2004; Madore et al., 2020; Rugg and Curran, 2007). The LPC old/new effect reflects a more positive amplitude for old items, compared to new items in the left parietal area, from 500 to 800 ms after stimulus onset (Curran and Friedman, 2004; Forester et al., 2019; Madore et al., 2020; Zhang et al., 2023a), indicative of enhanced recollection. Therefore, LPC can serve as a sensitive indicator of distinctiveness (Zhang et al., 2023a). The FN400 is an early negative component. The FN400 old/new effect reflects a more positive amplitude for old items compared to new items in the frontal area, from 300 to 500 ms after stimulus onset, indicative of enhanced familiarity. Therefore, FN400 can serve as an indicator of strength (Mecklinger and Jäger, 2009; Zhang et al., 2023a).

Zhang et al. (2023a) used a block design to investigate how reading aloud affects recollection and familiarity at the ERP level. At the behavioral level, they found that reading aloud can enhance both recollection and familiarity simultaneously. However, the ERP results showed a significant PE only in the LPC old/new effect, with no significant PE in the FN400 old/new effect. Simultaneously, they used multivariate pattern analysis (MVPA) to decode the time-course of neural activity for studied trials compared to new trials (aloud vs. new and silent vs. new) during the recognition phase. The results showed that stable decoding could only be achieved after 500 ms, which is consistent with the LPC effect (Zhang et al., 2023a). These findings indicated that reading aloud only enhanced recollection compared to silent reading, with no evidence of enhanced familiarity. They suggested that the evidence of familiarity-related PE observed at the behavioral level may reflect weak recollection.

The above results merely support the notion that recollection/distinctiveness contributes to the PE. Nevertheless, Zhang et al. (2023a) used a block design. As mentioned earlier, in mixed-lists the potential costs may lead to an amplification of the PE in familiarity (Ozubko et al., 2020). Therefore, we speculate that although Zhang et al. (2023a) did not observe a PE on the FN400 old/new effect in a block design, the presence of potential costs in mixed-lists may amplify the advantage of reading aloud over silent reading on the FN400 old/new effect, thereby leading to a significant PE on the FN400 old/new effect. This indicates that familiarity/strength may contribute to the PE in mixed-list designs rather than in block designs. Investigating this issue is of great importance for understanding the mechanisms of the PE (i.e., the roles of distinctiveness and strength). At the same time, developing an understanding of how reading aloud influences basic recognition memory processes (recollection and familiarity) is crucial for constructing a comprehensive modern theoretical framework for the PE in the future.

Overall, the purpose of this study was to examine the effect of reading aloud on recollection and familiarity in mixed-lists using ERP technology. We posit that if reading aloud enhances recollection rather than familiarity, this suggests that the PE is a function of distinctiveness rather than strength, and that the mechanism of the PE is common and not dependent on a blocked paradigm. Also, If reading aloud could enhance both recollection and familiarity, compared to silent reading, it indicates that in mixed-lists PE relies on both distinctiveness and strength, and the mechanism of PE is potentially unique to the paradigm or task. Furthermore, this study attempts to use MVPA to decode the temporal dynamics of the PE during the recognition phase (aloud vs. silent).

## 2 Materials and methods

The study was conducted in compliance with Good Clinical Practice and the Declaration of Helsinki and this study was approved in 2024 by the Human Research Ethics Committee at Flinders University, project no. 6844. Written informed consent was obtained from all participants.

### 2.1 Participants

We recruited 35 participants at Southwest University (China) and tested them. Due to high artifacts, 5 participants were excluded, leaving 30 participants (M_age_ = 22.2, SD = 3.7; 20 female) for the final analysis. A post hoc sensitivity analysis indicated that testing 30 participants with α = 0.05, a power of 0.8, and default parameters would allow us to detect a medium effect size in our paired-sample *t*-test (Effect size *dz* = 0.53).

### 2.2 Stimuli

To align as closely as possible with the classic mixed-list design, we maintained the same number of vocabulary items as in previous classic studies (Fawcett and Ozubko, 2016). We selected 240 frequently used two-character nouns from the vocabulary database of Zhang et al. (2023a) (familiarity: *M* = 5.4, SD = 1), with frequency ranging from 1 to 5,848 occurrences per million (*M* = 593, SD = 884) according to SUBTLEX-CH and CNCORPU (Cai and Brysbaert, 2010; Xiao, 2016). These items were subdivided into two sets matched for familiarity; one set was studied and the other served as the new items on the recognition test.

### 2.3 Experimental procedure

**Figure 1:**
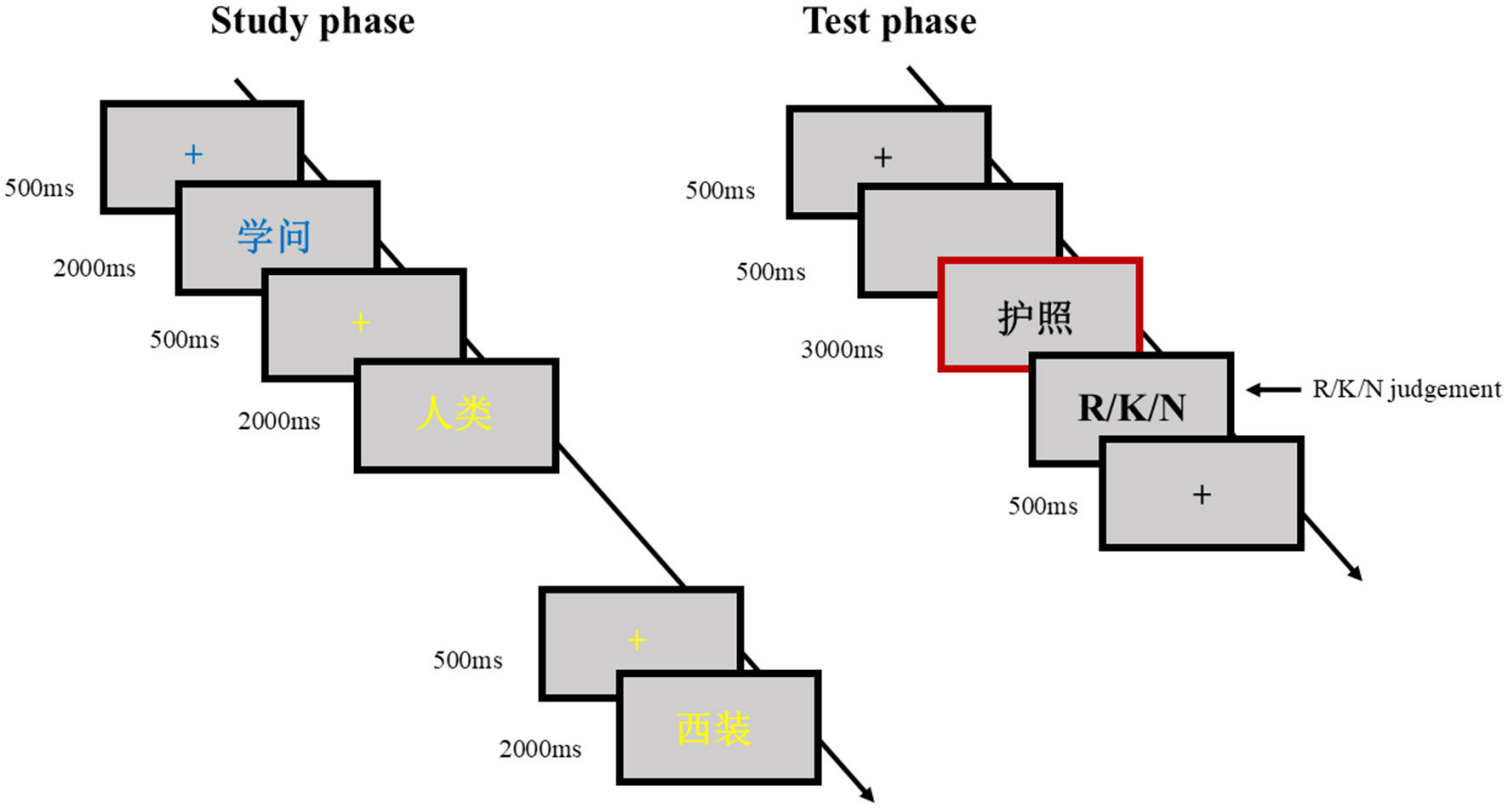
Flowchart of this study. During the study phase, two colors of fonts appeared randomly mixed. Afterward, participants entered the test phase, and ERP data were recorded during the stimulus stage indicated by the red box.

This study follows a within-subject design with 2 conditions (Memory condition: reading aloud, silent reading) to explore how different memory condition affect recollection and familiarity in recognition. During the study phase, in the mixed-list design, a total of 60 words were read aloud, and 60 words were read silently. Words were presented in either yellow or blue to indicate which action participants should take. For half of the participants, yellow indicated silent reading, and blue indicated reading aloud. These instructions were reversed for the remaining participants, and the items in the study phase were randomly mixed. During the study phase, participants first saw a 500 ms fixation point, followed by a 500 ms blank screen, and then a 2,000 ms presentation of words (2.65° × 1.30° visual angle). After the study phase, participants entered the test phase. During this phase, the words were presented in black on a gray background. Adequate breaks were scheduled in between. Participants were then presented with 60 read-aloud words, 60 silently read words, and 120 new words. Specifically, participants first saw a word that remained fixed on the screen for 3 s. Following this, they proceeded to an R/K/N judgment task. They were required to categorize the words as “remember,” “know,” or “new.” “Remember” was to be used if participants recalled contextual details from when they encoded the word (e.g., what they were thinking about or felt when they saw the item and what items had come before or after it). “Know” was to be used if they recognized having seen the word before but could not recall contextual details. “New” was to be used if they were certain they hadn't encountered the word before or were unsure if they had (Figure 1).

### 2.4 Statistical analysis

We used SPSS for the data analysis. Effects were deemed significant when *p* < 0.05. We conducted paired-samples *t*-tests on the behavioral data. For the ERP data, a three-factor repeated measures ANOVA was first performed, followed by paired-samples *t*-tests. Estimates of effect size are provided for significant comparisons using partial-eta squared ($\eta_{\text{p}}^{2}$) for ANOVAs and Cohen's d for *t*-tests.

### 2.5 ERP data recording

Participants were tested in a dimly lit, soundproof room. EEG data were recorded using a 64-channel Brain Products system (Brain Products GmbH, Germany; passband: 0.01–100 Hz) with tin electrodes mounted on a standard elastic cap based on the international 10–20 system. Data were analyzed using MATLAB and its toolbox EEGLAB. Electrodes were referenced to electrode FCz; offline data reference to the mean value of left and right mastoid. The electrodes on the outside of the right eye were used to monitor horizontal eye movement, and the electrodes on the underside of the left eye were used to monitor vertical eye movement. EEG was recorded at a sample rate of 500 Hz. Electrode impedances were maintained below 5 kΩ during data recording. Data were filtered with a 0.1–30 Hz bandpass filter, followed by independent component analysis (ICA) for each participant to identify and discard eyeblink-related components. Trials with voltages over −100 μV to +100 μV were removed. And EEG data were recorded during in test phases, ERPs were extracted during the item presentation of test phase.

As in previous studies, we focused on analysis of the frontal old/new effect and parietal old/new effect (Jäger et al., 2006; Mecklinger and Jäger, 2009; Zhang et al., 2023a). The LPC and FN400 were measured during the test phase. For the FN400 old/new effect, we selected 2 electrode points on the frontal region (F1, F3); for the LPC old/new effect, we selected 2 electrode points in the left parietal region (P1, P3) (Forester et al., 2019; Madore et al., 2020; Zhang et al., 2023a). For each component, we calculated the average amplitude across the indicated electrode sites.

### 2.6 Multivariate pattern analysis

MVPA was used to decode the time-course of word recognition in the recognition phase (test phase). MVPA is a multivariate analytical technique and is typically used when referring to the practice of characterizing (decoding) the difference between experimental conditions based on their patterns of brain responses (Fahrenfort et al., 2018). MVPA has been demonstrated to be a superior method to understand the nature of memory (Xue, 2018). MVPA involves training a classifier (a pattern classification algorithm) to distinguish different patterns of brain activity associated with different experimental variables of interest, which is more sensitive than conventional ERP analysis. This is because MVPA uses whole-brain activity to depict neural activity patterns over time (Li et al., 2022, 2024; Sharifian et al., 2021), and because the neural stability of decoding performance can be analyzed (King and Dehaene, 2014). To this end, we performed MVPA on the pre-processed EEG data using the Amsterdam decoding and modeling (ADAM) toolbox (Fahrenfort et al., 2018; Zhang et al., 2023a). MVPA involved a backward decoding classification algorithm (linear discriminant analysis). MVPA decoding captures differences between pairs of classes at the whole-brain level (i.e., aloud vs. new, silent vs. new, aloud vs. silent). Before performing MVPA, the EEG data were down-sampled offline to 50 Hz to facilitate decoding (Fahrenfort et al., 2018; Zhang et al., 2023a). The EEG data at individual electrode channels were used as classification features, and all electrodes were used to create features.

In terms of MVPA analysis, the classifier at each time point was trained and tested, using 5-fold cross-validation, to minimize potential biases resulting from the assignment of trials to different groups. We performed 5 iterations of the entire procedure, shuffling the order of the trials at the beginning of each iteration (Li et al., 2024). Based on the guidelines provided by Fahrenfort et al. (2018), after exporting the MVPA files, we utilized the scripts from Fahrenfort et al. (2018) to perform classification analysis (decoding) and temporal generalization analysis (All analysis scripts can be referred to in: Fahrenfort et al., 2018). To measure the classification performance (decoding), we used the area under the curve (AUC) as a metric, a larger AUC value means better classification performance (Fahrenfort et al., 2018). An AUC value of 0.5 indicated chanced classification performance. The results were corrected for multiple comparisons by cluster-based permutation tests (*p* < 0.05; 1,000 iterations). Finally, stability of classified neural activity over time was detected using temporal generalization analysis (King and Dehaene, 2014). This analysis explored whether the decoded neural activity patterns were stable by training the classifier at each time point and testing the classifier at all time points (Fahrenfort et al., 2018). The temporal generalization matrix was then obtained. As a result, if the classification accuracy outside the diagonal axis was above the chance level it indicated stable neural activity (Fahrenfort et al., 2018; Li et al., 2022, 2024; Zhang et al., 2023a).

## 3 Results

### 3.1 Behavioral data

**Figure 2:**
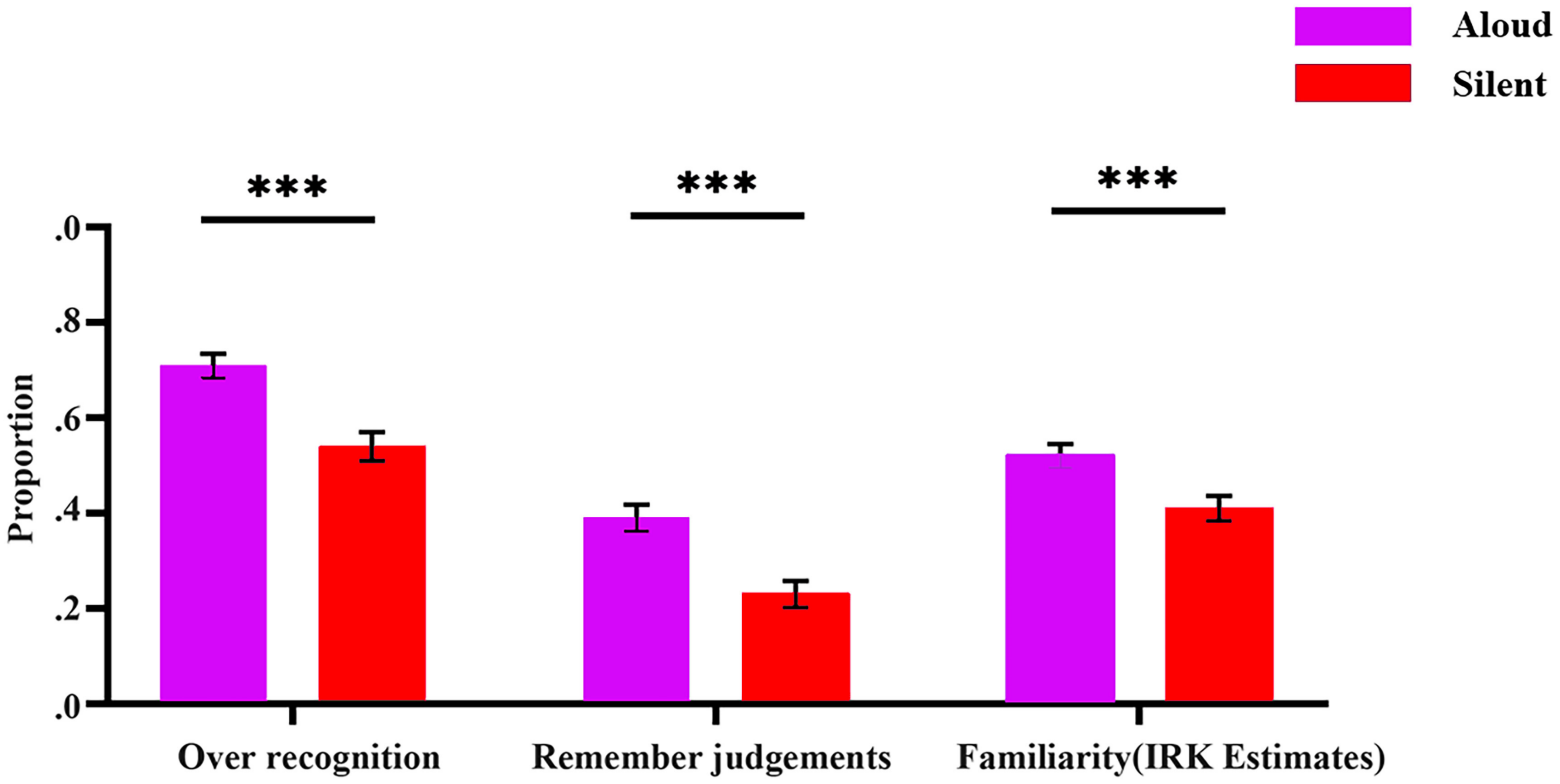
Behavioral data: overall recognition, recollection, and familiarity by condition. Error bars indicate the standard error of each mean. ***p < 0.001.

Our behavioral measures were analyzed in paired-samples *t*-test with condition (aloud, silent) as the factor: (1) overall recognition (percentage of items correctly identified as “old” at test; i.e., sum of remember + know judgments), (2) remember judgments (percentage of old items to which participants made a “remember” response), and (3) “know” judgments, and (4) familiarity estimates (as defined below). Table 1 provides the means and Figure 2 displays them.

**Table 1:** Behavioral data: overall recognition, recollection, and familiarity by condition.

	**Aloud**		**Silent**		***F*(1, 29)**	*** $\eta_{p}^{2}$ ***
**Measure**	**M**	**SD**	**M**	**SD**		
Overall recognition	0.71	0.11	0.54	0.14	66.71^*^	0.69
Recollection (R judgments)	0.39	0.18	0.23	0.15	55.24^*^	0.66
K judgments	0.31	0.1	0.3	0.11	0.23	0.008
Familiarity (IRK estimates)	0.52	0.13	0.4	0.14	21.43^*^	0.425

R, remember; K, know; IRK, independence remember-know. ^*^p < 0.05.

Overall recognition was greater in the aloud condition than in the silent condition, *t*(29) = 8.168, *p* < 0.001, Cohen's *d* = 0.63, thus demonstrating a robust PE on recognition.

In terms of the rate of remember judgments, as was true for overall recognition, remember judgments were more common in the aloud condition than in the silent condition, *t*(29) = 7.433, *p* < 0.001, Cohen's *d* =2.53.

The rate of know (K) judgments was similar across the conditions, *t*(29) = 0.481, *p* = 0.634, Cohen's *d* = 0.09. However, in the remember/know task, the rate of K judgment underestimates familiarity because participants who experience familiarity will only report a K judgment if recollection fails—otherwise they will report a remember (R) judgment (Yonelinas, 2002; Yonelinas et al., 2010). To compensate for this underestimation, familiarity was estimated using the independence remember-know (IRK) correction K/(1-R) (Table 1; see Yonelinas and Jacoby, 1995). Estimates of familiarity were greater in the aloud condition than in the silent condition, *t*(29) = 4.42, *p* < 0.001, Cohen's *d* = 3.796.

### 3.2 ERP data

#### 3.2.1 Recognition phase: LPC effect (500–800 ms)

**Figure 3:**
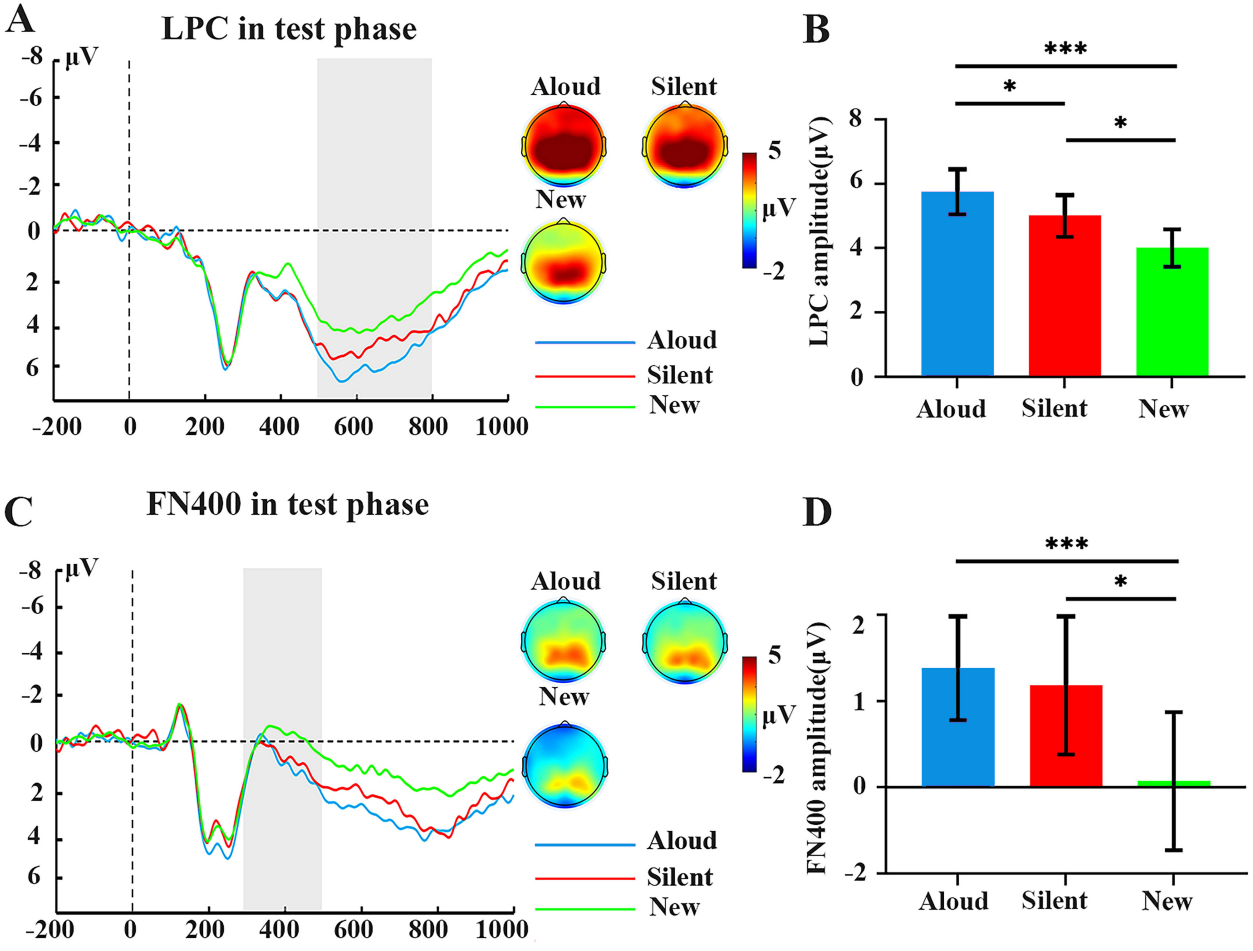
Amplitude of LPC and FN400 (Test Phase) ERP components by condition. **(A)** Waveforms and topography of LPC (test phase). **(B)** Evoked LPC amplitudes (test phase). **(C)** Waveforms and topography of FN400 (test phase). **(D)** Evoked FN400 amplitudes (test phase). **(A, C)** Shaded regions represent the defined time windows. **(B, D)** Error bars indicate the standard error of each mean. *p < 0.05 and ***p < 0.001.

To evaluate the possible contribution of recollection to the PE, we compared mean LPC amplitude during the test phase across the parietal region electrodes (P1, P3; Figure 3) for hits to aloud and silent trials, and for correct rejections of new trials. The ANOVA revealed a significant main effect of condition, *F*(2,58) = 8.82, *p* < 0.001, η_p_^2^ = 0.233. The LPC amplitude was greater on aloud test trials than on new trials, *p* = 0.001, Cohen's *d* = 1.14, showing a significant LPC old/new effect (difference) for aloud trials. The LPC amplitude was greater on aloud test trials than on silent trials, *p* = 0.032, Cohen's *d* = 0.47, showing that the LPC old/new effect for aloud trials is greater than for silent trial. LPC amplitude was greater on silent test trials than on new trials, *p* = 0.037, Cohen's *d* = 1.14, showing a significant LPC old/new effect (difference) for silent trials (Table 2 and Figure 3).

**Table 2:** ERP data: LPC, and FN400.

**Components**	**Aloud**		**Silent**		**New**		***F*(2, 58)**	** $\eta_{p}^{2}$ **
	**M**	**SD**	**M**	**SD**	**M**	**SD**		
LPC	5.75	3.95	5	3.6	4.03	3.2	8.82^*^	0.23
FN400	1.38	4.2	1.18	4.5	0.06	4.3	6.45^*^	0.18

The unit of the data is “microvolts” (μV). ^*^p < 0.05.

#### 3.2.2 Recognition phase: FN400 effect (300–500 ms)

To evaluate the possible contribution of familiarity to the PE, we compared mean FN400 amplitude during the test phase across the frontal region electrodes (F1, F3; Figure 3). The ANOVA revealed a significant main effect of condition, *F*(2,58) = 6.45, *p* = 0.003, η_p_^2^ = 0.182. The FN400 amplitude was more positive for hits to aloud test items than for correct rejections of new test items, *p* < 0.001, Cohen's *d* = 0.782, the latter showing a significant FN400 old/new effect. The FN400 amplitude was more positive for hits to silent test items than for correct rejections of new test items, *p* = 0.017, Cohen's *d* = 0.782, the latter showing a significant FN400 old/new effect. However, aloud trials, silent trials were equivalent, *ps* > 0.05, showing no difference in FN400 old/new effect of the two memory conditions. Thus, no PE on familiarity was found using this measure (Table 2 and Figure 3).

### 3.3 Multivariate pattern analysis

**Figure 4:**
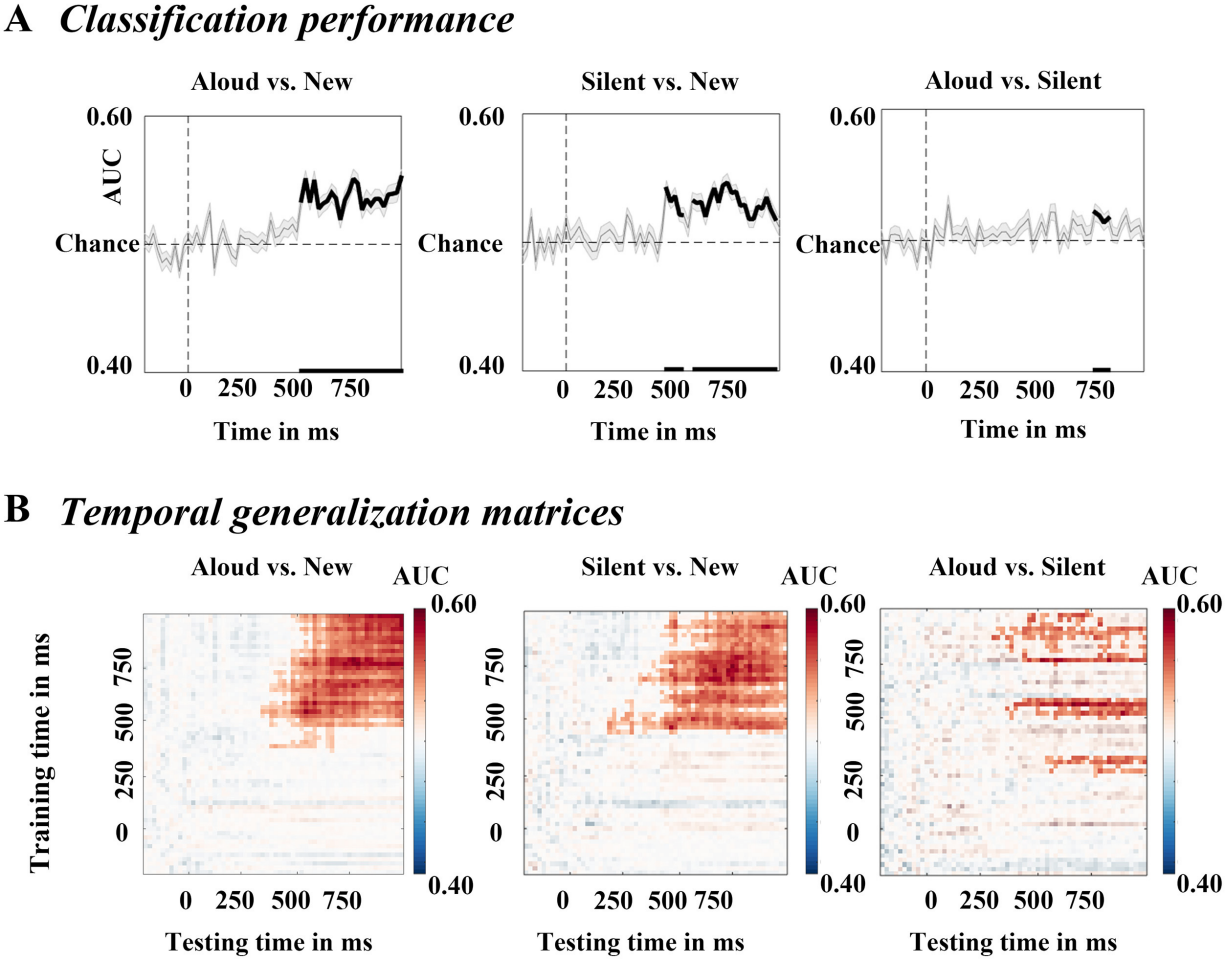
Mean classification performance and spatio-temporal decoding in test phase. **(A)** Spatio-temporal decoding of old/new contrast result in recognition. Classifier accuracy was threshold (cluster-based correction, *p* < 0.05). Gray lines indicate classifier accuracy. Solid black lines indicate significant clusters. Gray shaded contours in classification performance plots represent standard error of the mean. **(B)** Temporal generalization matrices. Saturated colors indicate significant samples (*p* < 0.05).

We observed significant classification for each pair of conditions using MVPA. MVPA revealed a significant above-chance difference in aloud vs. new classification from 540 to 1000 ms post-stimulus onset (paired *t*-test, *p* < 0.05, cluster corrected), in silent vs. new classification from 480 to 580 ms and from 600 to 980 ms post-stimulus onset (paired *t*-test, *p* < 0.05, cluster corrected), and for aloud vs. silent classification from 760 to 840 ms post-stimulus onset (paired *t*-test, *p* < 0.05, cluster corrected) (Figure 4A).

Because significant decoding was found in all three comparisons, we next calculated temporal generalization matrices to test the stability of neural activity patterns with underlying significant classification performance (Li et al., 2022). A cluster of significant above-chance activity was stable and significant from 380 to 1000 ms post-stimulus onset for the aloud vs. new comparison, from 140 to 980 ms and from 260 to 1000 ms post-stimulus onset for the silent vs. new comparison, and from 440 to 980 ms and from 520 to 1000 ms post-stimulus onset for the control vs. new comparison. The time generalization matrices indicated that the neural activity could be decoded by the trained classifiers during these time windows, suggesting that the differences between pairs of conditions were stable over time (Figure 4B).

## 4 Discussion

PE is the phenomenon where the memory of reading aloud is better than that of silent reading. To date, few studies have investigated PE using neuroimaging techniques (Bailey et al., 2021; Hassall et al., 2016; Tan et al., 2022; Zhang et al., 2023a). To further examine the mechanism of the PE, our study is the first to systematically investigate the impact of reading aloud on recollection and familiarity in a mixed-list design and to explore whether the mechanism underlying recognition PE is general or task-specific. Theoretically, the PE on recollection is consistent with reading aloud enhancing distinctiveness, while the PE on familiarity is consistent with reading aloud enhancing memory strength (Bailey et al., 2021; Fawcett and Ozubko, 2016; Ozubko et al., 2012). This study extracted the EEG amplitude during the testing phase and the results showed that, behaviorally, reading aloud increased overall recognition, recollection, and familiarity compared to silent conditions. At the ERP level, the larger LPC ERP component for aloud items further confirmed that reading aloud enhanced memory by increasing recollection. However, the FN400 ERP component findings did not provide convincing evidence that reading aloud also enhanced familiarity (Figure 3). Furthermore, our MVPA decoding analysis of the PE revealed that classification between study trials and new trials could be accurately determined from about 500 ms of onset, suggesting that participants rely more on recollection to recognize aloud and silent words. Further, for the first time, we found significant decoding between aloud trials and silent trials at 760–840 ms, expanding previous research and supporting the role of recollection in the PE (Zhang et al., 2023a). Our results indicates that the mechanism of recognition-based PE is general rather than task-specific, with enhanced recollection/distinctiveness contributing to the recognition PE. We have discussed the results in the context of postulations from traditional theories and in the context of a new model based on our study results. These discussions provide important insight for the development of future research. Below we unpack these findings in more detail.

### 4.1 Effect of production on LPC at retrieval

Recollection is widely believed to be a process of controlled retrieval, which refers to memory based on contextual information (Rugg and Curran, 2007; Schaefer et al., 2011). The LPC old/new effect is considered a marker of recognition based on recollection (Bridger and Mecklinger, 2012). In the time window of 500–800 ms in this study, a significant LPC old/new effect was observed between both reading aloud and silent reading conditions and the correct rejection of new trials, which indicates that both reading aloud and silent reading produced recollection-based memory. This result is inconsistent with findings of Zhang et al. (2023a) that only discovered a significant LPC old/new effect in the aloud condition. There are some differences between this study design and that of Zhang et al. (2023a). Zhang et al. (2023a) included three studying conditions and a total of 240 words, compared to two conditions and a total of 120 words in the current study. Having fewer words to memorize in the study phase might result in higher memory clarity for silent reading, enabling the recall of some background information during recognition, thus causing a significant LPC old/new effect of silent reading.

Next, we compared the LPC old/new effect of reading aloud with that of silent reading. The results indicated a PE in the LPC old/new effect, suggesting that although silent reading activated recollection-based recognition memory, reading aloud remained superior in terms of recollection indicator. These results revealed the mechanism of PE, indicating that PE originates from the advantage of reading aloud over silent reading with respect to recollection/distinctiveness, supporting the distinctiveness account. These results demonstrated that, regarding recollection, the format or length of the lists studied by participants might not influence the mechanism of PE, suggesting consistency and generality in the PE mechanisms across different paradigms/lists (Zhang et al., 2023a). Subsequently, we discuss the familiarity in the mixed-list.

### 4.2 Effect of production on FN400 at retrieval

In comparison to recollection, familiarity reflects an automated extraction process that lacks contextual information about one's encoding (Migo et al., 2012; Zhang et al., 2023a). According to the explanation of strength account, reading aloud enhances participants' familiarity with words thus providing a memory advantage over silent reading. At the behavioral level, the current study assessed familiarity using the corrected familiarity indicator (Ozubko et al., 2012; Fawcett and Ozubko, 2016; Zhang et al., 2023a). We found a familiarity-based PE in the mixed-list design, which is consistent with the strength account (e.g., Bodner and Taikh, 2012); however, there was no significant PE in the K judgments.

In terms of ERP, the FN400 old/new effect has been postulated to reflect familiarity-based recognition (Rugg and Curran, 2007; Wang et al., 2021). Firstly, we found that, like the LPC old/new effect, there is a significant FN400 old/new effect in both the reading aloud and silent reading conditions. This indicates that both reading aloud and silent reading activate familiarity-based recognition. Zhang et al. (2023a) found a significant FN400 old/new effect only in the reading aloud condition. We argue that the difference between the two study results is related to the number of vocabulary items memorized. Given that the memory for silent reading is weak, it is not surprising that reducing the number of items would allow participants to generate more familiarity.

The main aim of this section is to discuss whether there is a significant PE in the FN400 old/new effect. Our hypothesis was that in the mixed-list, due to the potential cost of silent reading, familiarity-based recognition during silent reading will be further reduced. As a result, the advantages of familiarity in the aloud condition will be amplified; thus, we will observe a PE in FN400 old/new effect. However, the current results did not support the above hypothesis. Although we found a significant FN400 old/new effect in both reading aloud and silent reading conditions, there was no difference between the two. This aligns with the findings of Zhang et al. (2023a). The current results and previous results indicate that there is no FN400 old/new effect PE in either mixed-list or block designs. Therefore, the advantage of familiarity at the behavioral level might only be a form of “weak recollection” (Wixted et al., 2010; Zhang et al., 2023a).

Even though the FN400 is currently considered to be the best indicator of familiarity and is generally accepted (Madore et al., 2020; Rugg and Curran, 2007), it might also reflect semantic processing of words or perhaps something broader (Voss and Federmeier, 2011; Leynes and Mok, 2017). Therefore, future research should explore if another ERP component could index familiarity-based recognition more effectively.

In addition, researchers should explore the impact of reading aloud on recollection and familiarity in a between-subjects design in future studies. This is because the PE of recollection may only emerge in within-subjects designs, while the between-subjects PE may rely more on strength/familiarity (Bodner et al., 2020; Fawcett and Ozubko, 2016; Whitridge et al., 2024).

### 4.3 Insights from MVPA decoding

Our research decoded EEG data during the test phase in a mixed-list or event-design memory task using MVPA technology. MVPA is a crucial method for researchers to understand the nature of memory (Xue, 2018). In terms of decoding between the study conditions (aloud and silent) and new trials, significant decoding was observed only after 500 ms, not before, regardless of whether it was the reading aloud condition with good memory or the silent reading condition. This is consistent with the LPC time window rather than the FN400. This pattern is consistent with the ERP results and previous decoding findings, indicating that recognition memory may rely more on recollection than familiarity. It also aligns with the ERP results, supporting the potential role of the LPC effect in the PE (cf. Curran et al., 2006; Zhang et al., 2023a).

Moreover, there has been significant interest in the temporal dynamics of different memory strategies (Forester et al., 2019; Pereira et al., 2021; Zhang et al., 2023a), which is crucial for understanding the core mechanisms of advantageous memory strategies. Until now, no research has decoded the temporal dynamics between different memory strategies (such as the time course between reading aloud and silent reading).

To further directly decode and unpack the differences between aloud trials and silent trials, we are the first to decode the time course of two memory strategies (aloud vs. silent). This is important for understanding the mechanisms of the PE, as PE fundamentally involves a comparison between reading aloud and silent reading. The results showed significant classification between reading aloud and silent reading in later time windows (corresponding to the LPC time window). This demonstrates that increased distinctiveness/recollection plays a significant role in the PE, while strength/familiarity does not. This finding is consistent with our ERP results. The two strategies could be decoded in the later time window, further indicating that the enhancement of recollection has the potential to become a major mechanism underlying certain superior memory strategies, such as production. Future research should explore this possibility further.

### 4.4 The common mechanism of PE

This study investigated the effect of recollection and familiarity in mixed-list PE. Previous studies indicated that the PE of mixed-list designs is larger than that of block designs due to the costs of silent reading generated in mixed-lists (Bodner et al., 2014). Ozubko et al. (2020) found that, behaviorally, the cost was reflected in both recollection and familiarity, but Zhang et al. (2023a) showed EEG data in block design suggesting that reading aloud only enhanced recollection. We addressed two possibilities for PE. Firstly, regardless of design (blocked/mixed list) there is a common mechanism of PE that works through distinctiveness/recollection rather than strength/familiarity (MacLeod et al., 2022; Zhang et al., 2023a). Another possibility is that mixed-list PE depends on familiarity. Silent reading might incur a cost in mixed-lists, possibly amplifying the advantage of reading aloud in terms of familiarity and thereby producing a significant PE on the FN400 old/new effect. This implies that familiarity/strength might contribute to mixed-list PE, indicating that PE could be specific to the paradigm. The above inference produces a crucial question: Is the mechanism of PE specific to the paradigm or does it have common mechanisms? Collectively, they suggest that, studying the mechanisms of mixed-list PE is of great significance.

The current study revealed that in mixed-list designs, despite the possibility of amplifying the advantage of reading aloud on familiarity relative to silent reading, only a recollection-related PE was present. Also, our study decoded the time-course of PE (aloud trials vs. silent trials) for the first time. The current results suggest that in the test phase, trials of reading aloud and silent reading could only be decoded after 500 ms, which is consistent with the ERP results. The results of the mixed-list design is consistent with Zhang et al. (2023a), indicating that PE relies solely on recollection/distinctiveness rather than familiarity/strength. The basis of PE is broad and stable, at least within subjects.

### 4.5 Traditional explanation and feature space theory

This study aims to further explore the effects of reading aloud on recollection and familiarity, building upon Zhang et al. (2023a). Understanding the processes of recollection and familiarity is crucial for constructing a modern theoretical framework for the mechanisms underlying the PE. Consistent with previous research, this study posits that the PE in recollection is driven by distinctiveness, and the results align with the predictions of the distinctiveness account (Fawcett and Ozubko, 2016; Zhang et al., 2023a).

However, recent years have seen the emergence of several models. A memory effect is often complex (Hourihan and Fawcett, 2024; Kelly et al., 2024). For example, the (Retrieving Effectively from Memory framework) REM model suggests that recognition-based PE may stem from longer retrieval times during the testing phase, while the model by Wakeham-Lewis et al. (2022) argues that PE arises from the enhancement of semantic encoding due to vocalization (Wakeham-Lewis et al., 2022). These models are closely tied to the distinctiveness account. However, they all emphasize that the PE may not be entirely a direct result of distinctiveness.

Here, the recently proposed Feature-Space Theory provides an important theoretical perspective challenging the distinctiveness account. MacLeod et al. (2010) suggested that reading aloud enhances the retention of distinctiveness factors, allowing participants to consciously recognize that they have read the word when they see it again, and matching the word through this awareness, which is similar to recollection. In contrast, the Feature-Space Theory emphasizes that the enhanced attention from reading aloud captures phonological features within a feature space. In the recognition phase, participants are thought to focus their attention on phonological features to determine whether the probe word is one that has been learned or is new. Items that have been produced tend to store more phonological features, making them more likely to form a match in memory compared to items that have been read silently. In the recognition phase, the view of Caplan and Guitard (2024) is more aligned with that of Kolers (1973), suggesting that the matching or recognition process during this phase should be unconscious.

The key difference between the theories of MacLeod et al. (2010) and Caplan and Guitard (2024) in the recognition phase lies in the fact that MacLeod et al. (2010) believes that retrieval is conscious. Caplan and Guitard (2024) tend to view retrieval as unconscious (Hourihan and Fawcett, 2024). This raises questions on whether recollection can be considered a slow process and an active retrieval (Yonelinas et al., 2022), and whether the Feature-Space Theory can directly predict the PE in recollection. Since the Feature-Space Theory is a mathematical model and did not attempt to predict the PE in recollection in the paper, we remain cautious about this. Since the LPC component, an indicator of recollection, is a slow and late positive ERP wave that typically reflects active retrieval (Guillaume and Tiberghien, 2013; Parks, 2007; MacKenzie and Donaldson, 2007; Yonelinas et al., 2022; Zhao et al., 2020), this does not align with the Feature-Space Theory which suggests that retrieval tends to be automatic. Nevertheless, this is consistent with the predictions of the distinctiveness account, which proposes that participants actively retrieve the words. Therefore, this study supports the role of distinctiveness in the PE and also reinforces the distinctiveness account. We tend to believe that PE recognition is based on active retrieval rather than automatic processing. Another piece of evidence supporting this comes from familiarity (FN400).

In terms of familiarity, familiarity is considered a relatively automated component of recognition, with fast processing speed. This explains why in everyday life, when we see someone, we may instinctively feel a sense of familiarity. Considering that the early frontal component FN400 is a fast and automated processing component, it also makes sense why FN400 is regarded as a representative component of familiarity (Guillaume and Tiberghien, 2013; Parks, 2007; MacKenzie and Donaldson, 2007; Yonelinas et al., 2022; Zhao et al., 2020). Feature-Space Theory suggests that matching or recognition is unconscious, which would imply a significant PE should be observed in the early components of fast, automatic processing. However, we did not observe a PE in the FN400 (familiarity). This is inconsistent with the hypothesis of unconscious matching proposed by Feature-Space Theory.

Furthermore, we examined evidence from MVPA, and if this process is primarily unconscious, we would expect to observe whole-brain decoding in MVPA during the early time window. This is because MVPA is more sensitive than ERP, allowing for the decoding of whole-brain activity across a broader spectrum, which helps avoid biases related to electrode selection and time window constraints. This approach more accurately reflects the true nature of memory processes (Xue, 2018). However, we did not observe decoding before 500 ms, which is also different from the predictions of unconscious matching or retrieval.

Feature-Space Theory, as an excellent mathematical model, has successfully predicted some effects of the PE. However, we are still unclear about how to predict the neural dynamics at the experimental level, how these dynamics interact with context-based recollection and familiarity, and how to anticipate the separations we observe in behavior and electrophysiology.

Future research should further explore these issues. Future studies should continue to test and advance these models by integrating computational models with experiments, moving beyond mere theoretical simulation to reveal what truly occurs for participants during the experimental process. Perhaps future integration of neuroscience techniques, experiments, and these models will be a good way to unravel the mystery.

Overall, this study supports the role of distinctiveness in the PE, rather than strength.

## 5 Conclusion

Previous studies detected the effect of reading aloud on recollection and familiarity at both the behavioral and EEG levels, with results indicating that reading aloud may enhance recollection rather than familiarity. The current study used EEG technology to systematically investigate, for the first time, the effects of reading aloud on recollection and familiarity in a mixed-list PE, thereby establishing a fundamental understanding of the PE. At the ERP level, the PE was observed only in the LPC old/new effect rather than the FN400 old/new effect, indicating that the PE exists only in recollection. This suggests that PE relies on distinctiveness rather than strength. At the MVPA level, in order to further understand the mechanisms of the PE, this study for the first time decoded the time-course of reading aloud and silent reading during the test phase, indicating that only EEG data after 500 ms could be decoded, consistent with the time window of the LPC component. Overall, the current study demonstrated that the mechanism of the PE may exhibit broad and stable cross-paradigm consistency. In the conclusion of the paper, this study discusses traditional theories and Feature Space Theory based on the findings of the research. We reported the inconsistency between our results and the unconscious processing hypothesis of Feature Space Theory. Furthermore, this study suggests that enhanced distinctiveness/recollection may be a shared mechanism underlying certain advantageous memory strategies.

## Data Availability

The raw data supporting the conclusions of this article will be made available by the authors, without undue reservation.
