# Corrigendum: EEG-based multivariate and univariate analyses reveal the mechanisms underlying the recognition-based production effect: evidence from mixed-list design

**DOI:** 10.3389/fnhum.2025.1601104

**Published:** 2025-04-25

**Authors:** Bohua Zhang, Alhassan Abdullah, Minmin Yan, Yongqing Hou, Antao Chen, Helen McLaren

**Affiliations:** ^1^College of Education, Psychology and Social Work, Flinders University, Adelaide, SA, Australia; ^2^Faculty of Psychology, Southwest University, Chongqing, China; ^3^School of Social Work and Arts, Charles Sturt University, Thurgoona, NSW, Australia; ^4^Department of Neurobiology and Department of Psychiatry of the Second Affiliated Hospital of Zhejiang University School of Medicine, School of Brain Science and Brain Medicine of the Zhejiang University School of Medicine, Hangzhou, China; ^5^School of Psychology, Shanghai University of Sport, Shanghai, China; ^6^School of Allied Health (VIC), Australian Catholic University, Melbourne, VIC, Australia

**Keywords:** reading aloud, silent reading, LPC, FN400, MVPA

In the published article, there was an error in affiliation(s) [5]. Instead of “School of Psychology, Shanghai Jiaotong University, Shanghai, China”, it should be “School of Psychology, Shanghai University of Sport, Shanghai, China”.

The authors apologize for this error and state that this does not change the scientific conclusions of the article in any way. The original article has been updated.

